# Supplementary material for: Access and enrollment in safety net programs in the wake of COVID-19: A national cross-sectional survey
Source: PLoS One. 2020 Oct 6;15(10):e0240080. doi: 10.1371/journal.pone.0240080 (PMC7537892; doi:10.1371/journal.pone.0240080)
Supplement: S1 File — (DOCX) [file pone.0240080.s001.docx]

AmeriSpeak Omnibus April 2020 WAVE 2.0420.2

START OF UMINN SHADAC QUESTIONS

INSERT ITEM TIMESTAMPS: TIME_UM1START, DATE_UM1START

[SHOW IF BLOCK 1 NOT SHOWN FIRST IN RANDOMIZATION ORDER]

[DISPLAY]

DISPLAY_UMS.

The next few questions are on another subject.

[MP]

UMS1.

Which, if any, of the following has happened to your employment as a result of the coronavirus?

[CAWI - remove bold] <i>*Select all that apply.*</i>
[CATI] SELECT ALL THAT APPLY.

CAWI RESPONSE OPTIONS – RANDOMIZE:

1. I was not employed at the onset of the coronavirus [SP] [ANCHOR]
2. I am on paid leave because my employer closed due to the coronavirus
3. I have lost my job due to the coronavirus
4. I have had my work hours cut due to the coronavirus
5. I have had my pay cut due to the coronavirus
6. I am working more hours due to the coronavirus
7. I am working from home due to the coronavirus
8. I have gotten a new job because of the coronavirus
9. I have retired from work due to the coronavirus [ANCHOR] [SP]
10. The coronavirus has not affected my job [ANCHOR] [SP]

CATI RESPONSE OPTIONS – RANDOMIZE:

1. You were not employed at the onset of the coronavirus [SP] [ANCHOR]
2. You are on paid leave because your employer closed due to the coronavirus
3. You have lost your job due to the coronavirus
4. You have had your work hours cut due to the coronavirus
5. You have had your pay cut due to the coronavirus
6. You are working more hours due to the coronavirus
7. You are working from home due to the coronavirus
8. You have gotten a new job because of the coronavirus
9. You have retired from work due to the coronavirus [ANCHOR] [SP]
10. The coronavirus has not affected your job [ANCHOR] [SP]

[SP]

UMS2.

As a result of the coronavirus, would you say that *currently:*

RESPONSE OPTIONS:

1. You are unable to pay for necessary expenses (such as, food, rent/mortgage, utilities)
2. It’s harder than usual to pay for necessary expenses (such as, food, rent/mortgage, utilities)
3. There’s been no effect on your ability to pay for necessary expenses (such as, food, rent/mortgage, utilities)

[SP]

UMS3.

What, if any, effect has the coronavirus had on your health insurance situation?

CAWI RESPONSE OPTIONS:

1. My health insurance through work ended
2. I canceled my health insurance to pay for other expenses
3. I was uninsured at the onset of the coronavirus and am still uninsured
4. There’s been no effect on my health insurance

CATI RESPONSE OPTIONS:

1. Your health insurance through work ended
2. You canceled your health insurance to pay for other expenses
3. You were uninsured at the onset of the coronavirus and are still uninsured
4. There’s been no effect on your health insurance

[SHOW IF UMS3=1]

[MP]

UMS4.

You said your insurance through work ended, did you:

[CAWI - remove bold] <i>*Select all that apply.*</i>
[CATI] SELECT ALL THAT APPLY.

RESPONSE OPTIONS:

1. Apply for COBRA (the program that allows a person to continue to keep their employer’s insurance if they pay the full premium)
2. Apply for coverage through a health insurance exchange or state-based marketplace
3. Apply for Medicaid or another state public health insurance program
4. Get added or will be added to a family member’s health insurance plan
5. Apply for another form of coverage
6. Decided not to apply for new coverage and will be uninsured

[MP]

UMS5.

Which, if any, of the following changes in your use of health care services (medical, dental or mental health) has happened to you as a result of the coronavirus?

[CAWI - remove bold] <i>*Select all that apply.*</i>
[CATI] SELECT ALL THAT APPLY.

CAWI RESPONSE OPTIONS:

1. I delayed seeking <i>*medical care for coronavirus symptoms*</i> because of worry about the costs
2. I delayed seeking needed <i>*medical or dental care*</i> (not for coronavirus) because of worry about the costs
3. I delayed seeking care for <i>*mental health needs*</i> because of worry about the costs
4. I delayed seeking <i>*medical, dental, or mental health*</i> care or canceled my appointment to avoid contact with other patients
5. My medical, dental, or mental health appointments were canceled by my providers due to the coronavirus
6. There’s been no effect on my use of health care services [SP]

CATI RESPONSE OPTIONS:

1. You delayed seeking <i>*medical care for coronavirus symptoms*</i> because of worry about the costs
2. You delayed seeking needed <i>*medical or dental care*</i> (not for coronavirus) because of worry about the costs
3. You delayed seeking care for <i>*mental health needs*</i> because of worry about the costs
4. You delayed seeking <i>*medical, dental, or mental health*</i> care or canceled your appointment to avoid contact with other patients
5. Your medical, dental, or mental health appointments were canceled by your providers due to the coronavirus
6. There’s been no effect on your use of health care services

[GRID 4,3; SP]

UMS6.

Are you aware of any of the following government programs?

GRID ITEMS:

1. Medicaid or another state health insurance program
2. Health insurance exchanges (healthcare.gov) or a state based marketplace
3. Unemployment insurance
4. Food pantry/free meals program
5. Housing or renters’ assistance programs
6. Food stamps (SNAP)
7. Temporary Assistance for Needy Families (TANF)

CAWI RESPONSE OPTIONS:

1. Yes, I’ve heard of this program
2. No

CATI RESPONSE OPTIONS:

1. Yes, you’ve heard of this program
2. No

[GRID 4,3; SP]

UMS7.

Do you receive assistance from one of these programs or have you applied for any of these programs?

GRID ITEMS:

1. Medicaid or another state health insurance program
2. Health insurance exchanges or state based marketplace
3. Unemployment insurance
4. Food pantry/free meals program
5. Housing or renters’ assistance programs
6. Food stamps (SNAP)
7. Temporary Assistance for Needy Families (TANF)

CAWI RESPONSE OPTIONS:

1. Yes, I received assistance from these programs prior to the coronavirus outbreak
2. Yes, I applied for or used this program since coronavirus
3. No, I have never received nor applied for this program

CATI RESPONSE OPTIONS:

1. Yes, you received assistance from these programs prior to the coronavirus outbreak
2. Yes, you applied for or used this program since coronavirus
3. No, you have never received nor applied for this program

[SP]

UMS8.

Would you say your health in general is excellent, very good, good, fair, or poor?

CAWI RESPONSE OPTIONS:

1. Excellent
2. Very good
3. Good
4. Fair
5. Poor

CATI RESPONSE OPTIONS:

1. EXCELLENT
2. VERY GOOD
3. GOOD
4. FAIR
5. POOR

[SP]

UMS9.

Do you have an illness, chronic disease, or chronic condition(s) that requires regular, ongoing medications, diets, appointments, treatments, or therapies at this time?

CAWI RESPONSE OPTIONS:

1. Yes
2. No
3. I don't know

CATI RESPONSE OPTIONS:

1. Yes
2. No
3. You don't know

[SP]

UMS10.

How worried are you about whether you can afford needed medical care if you or someone in your family contracts the coronavirus?

RESPONSE OPTIONS:

1. Very worried
2. Somewhat worried
3. Not too worried
4. Not worried at all

[SP]

UMS11.

How worried are you right now about not being able to pay medical costs for a serious illness or accident not related to coronavirus?

RESPONSE OPTIONS:

1. Very worried
2. Somewhat worried
3. Not too worried
4. Not worried at all

[MP]

UMS12.

Which of these activities describes how you have coped with stress recently.

[CAWI - remove bold] <i>*Select all that apply.*</i>
[CATI] SELECT ALL THAT APPLY.

CAWI RESPONSE OPTIONS:

1. I have not had any additional stress [SP]
2. I talk more to my friends and family on phone or video chat
3. I am drinking alcohol more frequently
4. I am smoking or vaping more frequently
5. I am eating more
6. I am eating more unhealthy foods
7. I am exercising more
8. I am exercising less
9. I am increasing my use of social media
10. I am decreasing my use of social media
11. I am not doing anything different [SP]

CATI RESPONSE OPTIONS:

1. You have not had any additional stress [SP]
2. You talk more to your friends and family on phone or video chat
3. You are drinking alcohol more frequently
4. You are smoking or vaping more frequently
5. You are eating more
6. You are eating more unhealthy foods
7. You are exercising more
8. You are exercising less
9. You are increasing your use of social media
10. You are decreasing your use of social media
11. You are not doing anything different [SP]

[SP]

UMS13.

The federal government recently passed a $2 trillion stimulus bill that will provide aid to Americans including payment of up to $1,200 for individuals and $2,400 for married couples.

[SPACE]

Are you aware of this cash benefit?

CAWI RESPONSE OPTIONS:

1. Yes
2. No

CATI RESPONSE OPTIONS:

1. YES
2. NO

[SP]

UMS14.

[CATI: READ INTRO TO QUESTION AGAIN ONLY IF NECESSARY]

The federal government recently passed a $2 trillion stimulus bill that will provide aid to Americans including payment of up to $1,200 for individuals and $2,400 for married couples.

[SPACE]

Are you eligible?

CAWI RESPONSE OPTIONS:

1. No, I am not (or don’t think I am) eligible
2. Yes, I am (or think I am) eligible

CATI RESPONSE OPTIONS:

1. No, you are not (or don’t think you are) eligible
2. Yes, you are (or think you are) eligible

[SHOW IF UMS14=2]

[SP]

UMS15.

[CATI: READ INTRO TO QUESTION AGAIN ONLY IF NECESSARY]

The federal government recently passed a $2 trillion stimulus bill that will provide aid to Americans including payment of up to $1,200 for individuals and $2,400 for married couples.

[SPACE]

What will be your top priority for how you will spend this money?

CAWI RESPONSE OPTIONS:

1. Help pay my mortgage or rent
2. Help pay for food for myself/family
3. Help pay for utilities (electricity, water, heat, gas, internet, etc...)
4. Help pay off credit card debt
5. Help pay for health insurance premiums
6. Help pay for needed medical care
7. Help pay for medical bills for care already received
8. Help pay off student loans
9. Help with car payments
10. Other, please specify: [TEXTBOX]

CAWI RESPONSE OPTIONS:

1. Help pay your mortgage or rent
2. Help pay for food for yourself/family
3. Help pay for utilities (electricity, water, heat, gas, internet, etc...)
4. Help pay off credit card debt
5. Help pay for health insurance premiums
6. Help pay for needed medical care
7. Help pay for medical bills for care already received
8. Help pay off student loans
9. Help with car payments
10. Other, please specify: [TEXTBOX]

[GRID 5,5;SP]

UMS16.

*Over the next 4 weeks,* how confident are you that you will be able to pay for the following items <i>*with or without*</i> receipt of the federal cash benefit of $1,200 for individuals and $2,400 for married couples?

GRID ITEMS:

1. Food for [CAWI:myself/family][CATI:yourself/family]
2. Utilities (electricity, water, heat, gas, internet, etc.)
3. Mortgage or Rent
4. Student loans
5. Car payments
6. Health Insurance Premiums
7. Needed Medical Care
8. Medical bills for care already received
9. Credit card debts
10. Other debts

RESPONSE OPTIONS:

1. Not at all confident
2. Not very confident
3. Somewhat confident
4. Very confident
5. Not applicable

[GRID 4,4;SP]

UMS17.

It is anticipated that in the next 12-18 months, a vaccine for coronavirus will be available. However, at least at first, there may not be enough to go around. Public health authorities must set guidelines about who gets the vaccine first. Please indicate the level of priority that should be given for each of the listed groups.

GRID ITEMS – RANDOMIZE:

1. Front-line medical care staff working with coronavirus patients
2. Essential workers who interact with the public (postal workers, grocery clerks, etc.)
3. People who are pregnant
4. Children (0-18 years old) with serious illness who are at high risk of dying from the coronavirus
5. People age 65 years and older at high risk of dying from the coronavirus
6. Middle-aged people with serious illness who are at high risk of dying from the coronavirus
7. Adults (19-64) at moderate risk of dying from the coronavirus
8. Children (0-18 years old) at moderate risk of dying from the coronavirus

RESPONSE OPTIONS:

1. Low Priority
2. Medium Priority
3. High Priority

[MP]

UMS18.

One topic of current discussion is whether or not the government should pay for any costs of coronavirus. In your opinion, what costs related to coronavirus should be covered by the government, or should the government not cover the costs?

[CAWI - remove bold] <i>*Select all that apply.*</i>
[CATI] SELECT ALL THAT APPLY.

RESPONSE OPTIONS:

1. Cost of testing for coronavirus for everyone
2. Cost of testing for coronavirus for only the uninsured
3. Cost of treatment including hospital stays for coronavirus for everyone
4. Cost of treatment including hospital stays for only the uninsured
5. Payments to help people with lost wages or income
6. Payments to help businesses recover after coronavirus closure
7. Government should not pay for any of the costs associated with coronavirus [SP]

END OF UMINN SHADAC QUESTIONS
