# Supplementary material for: Access and enrollment in safety net programs in the wake of COVID-19: A national cross-sectional survey
Source: PLoS One. 2020 Oct 6;15(10):e0240080. doi: 10.1371/journal.pone.0240080 (PMC7537892; doi:10.1371/journal.pone.0240080)
Supplement: Attachment — Submitted filename: plos one.docx [file pone.0240080.s002.docx]

This paper is a straightforward comparison of economic hardship and enrollment in safety net programs between those who experienced COVID-19-related job loss (defined as either complete job loss or a reduction in hours) versus those who didn’t. Data come from a special COVID-19 economic impact questionnaire that the authors had appended to the April 24-26 survey of the AmeriSpeak panel. The key results are: 1) 28% of the sample experienced job loss, 2) there were sizeable differences in program participation even before the pandemic between those who would eventually experience job loss and those who would not (i.e. job loss hit the lower end of the labor market harder), and 3) those differences became even larger after the job loss happened, 4) there were huge differences in subsequent priorities for stimulus check spending and ability to pay for basic needs. There is also an attempt to examine the influence of demographic characteristics on these results, though the estimates are generally too imprecise to be useful.

These results are unsurprising and the analyses are fairly simplistic (e.g. no attempt to leverage exogenous variation to identify the causal effects of job loss on the various outcomes, rather than just its associations). That said, the data source is novel and there is certainly value to quantifying the magnitudes of the associations. The more attention that can be brought to the need for a robust social safety net and continued stimulus during this time of crisis, the better.

Comments/suggestions:

1) I don’t see the value of the regressions in Table 5. As the authors themselves concede, the confidence intervals tend to be too wide for the results to be terribly informative. I would just drop this table, freeing up space for better exposition in the text and potentially also allowing the clunky Table 2 to be split into multiple exhibits (more on that next).

2) It took me a while to figure out exactly what’s going on in Table 2. The confusing part is that there’s really two very distinct questions being asked:

a) How different was baseline enrollment (i.e. pre-job-loss) between those who would eventually lose their jobs/have hours cut versus those who would not? If I understand correctly, the value of those results is in showing that the people who got laid off/had their hours cut were disproportionately “vulnerable” even prior to the pandemic.

b) How different was *new* enrollment during the pandemic between those who experienced job loss/reduction versus those who did not? This is more like the usual difference-in-difference-style question.

Since these are such distinct questions, I think it would be less confusing if they were treated more distinctly throughout the paper. For instance, split Table 2 into two separate tables, and better explain their distinctiveness in the abstract, intro, and body of the paper. To provide one example of how the distinction is currently murky, the abstract says, “Those who experienced COVID-19 job loss versus those who did not were significantly more likely to have applied or enrolled in >1 program … and also significantly more likely to specifically have enrolled in unemployment insurance … and SNAP.” More likely to have enrolled *when*? Nothing in that sentence specifies *new enrollment during the pandemic.* So it ends up reading as a continuation of the previous sentence about baseline differences.

3) One substantive critique is that the authors ignore the well-known issue of misreporting in survey-based program participation measures. While the critique applies to all programs, the literature on misreporting in SNAP is especially well-developed. A recent paper that provides a detailed discussion of that literature is <https://onlinelibrary.wiley.com/doi/abs/10.1002/soej.12364>

4) Given the rapidly changing nature of the pandemic, a survey from late April is already a bit dated. The authors acknowledge that “data collected in late April 2020 may not generalize to issues and concerns arising in more recent periods.” I appreciate the caveat but they should push harder here. If the results don’t generalize, then why do we care? At lease *some* argument needs to be made for generalizability.

5) p. 13: “There was no statistically significant difference in participation in at least one safety net program between people who subsequently experienced COVID-19 job loss than those not experiencing job loss (50.0% versus 37.7%, p=0.120).” I don’t like this sentence. The magnitude of that difference is large, and the p-value is close to the threshold … more nuance is needed.

6) I’d give some thought to whether “job loss” is the best term to describe the “treatment” group. It’s actually the combination of those who lost jobs completely and those who had their hours reduced – and the majority is the latter. It’s really more of an “adverse employment shock” group. Calling it “job loss” may be catchier but it is a recipe for being misquoted in the media.
